# Exercise gas exchange in continuous-flow left ventricular assist device recipients

**DOI:** 10.1371/journal.pone.0187112

**Published:** 2018-06-01

**Authors:** Alessandro Mezzani, Massimo Pistono, Piergiuseppe Agostoni, Andrea Giordano, Marco Gnemmi, Alessandro Imparato, Pierluigi Temporelli, Ugo Corrà

**Affiliations:** 1 Exercise Pathophysiology Laboratory, Cardiac Rehabilitation Division, Scientific Institute of Veruno IRCCS, Istituti Clinici Scientifici Maugeri Spa SB, Veruno (NO), Italy; 2 Centro Cardiologico Monzino IRCCS and Department of Clinical Sciences and Community Health, Cardiovascular Section, University of Milan, Milan, Italy; 3 Bioengineering Service, Scientific Institute of Veruno IRCCS, Istituti Clinici Scientifici Maugeri Spa SB, Veruno (NO), Italy; 4 Echocardiography Laboratory, Cardiac Rehabilitation Division, Scientific Institute of Veruno IRCCS, Istituti Clinici Scientifici Maugeri Spa SB, Veruno (NO), Italy; Universita degli Studi di Napoli Federico II, ITALY

## Abstract

Exercise ventilation/perfusion matching in continuous-flow left ventricular assist device recipients (LVAD) has not been studied systematically. Twenty-five LVAD and two groups of 15 reduced ejection fraction chronic heart failure (HFrEF) patients with peak VO_2_ matched to that of LVAD (HFrEF-matched) and ≥14 ml/kg/min (HFrEF≥14), respectively, underwent cardiopulmonary exercise testing with arterial blood gas analysis, echocardiogram and venous blood sampling for renal function evaluation. Arterial-end-tidal PCO_2_ difference (P(a-ET)CO_2_) and physiological dead space-tidal volume ratio (VD/VT) were used as descriptors of alveolar and total wasted ventilation, respectively. Tricuspid annular plane systolic excursion/pulmonary artery systolic pressure ratio (TAPSE/PASP) and blood urea nitrogen/creatinine ratio were calculated in all patients and used as surrogates of right ventriculo-arterial coupling and circulating effective volume, respectively. LVAD and HFrEF-matched showed no rest-to-peak change of P(a-ET)CO_2_ (4.5±2.4 vs. 4.3±2.2 mm Hg and 4.1±1.4 vs. 3.8±2.5 mm Hg, respectively, both p >0.40), whereas a decrease was observed in HFrEF≥14 (6.5±3.6 vs. 2.8±2.0 mm Hg, p <0.0001). Rest-to-peak changes of P(a-ET)CO_2_ correlated to those of VD/VT (r = 0.70, p <0.0001). Multiple regression indicated TAPSE/PASP and blood urea nitrogen/creatinine ratio as independent predictors of peak P(a-ET)CO_2_. LVAD exercise gas exchange is characterized by alveolar wasted ventilation, i.e. hypoperfusion of ventilated alveoli, similar to that of advanced HFrEF patients and related to surrogates of right ventriculo-arterial coupling and circulating effective volume.

## Introduction

Continuous-flow left ventricular assist devices (LVAD) are increasingly used as destination therapy in end-stage reduced ejection fraction chronic heart failure (HFrEF) patients [1–3]. However, most studies show that last generation devices have modest effects on functional capacity [4–8]. This is due to both the severe functional impairment of patients prior to LVAD implantation and the scarce ability of the fixed-speed LVAD/dysfunctioning native left ventricle combination to cope with increasing preload during exercise [9,10]. As a consequence, not only have training programs of LVAD recipients been implemented [11], but also devices able to adapt pump-generated blood flow to exercise energetic demands have been conceived [12,13]. Quite surprisingly, however, gas exchange dynamics and ventilation/perfusion matching during exercise [14–16] have not been systematically evaluated as yet in LVAD recipients.

The aim of this study was to compare respiratory and arterial blood exercise gas exchange of a group of LVAD-implanted patients with those of two groups of HFrEF patients with similarly reduced and significantly higher peak volume of oxygen uptake (peak VO_2_), respectively. Due to the inability of LVAD to significantly increase systemic blood flow in response to exercise [8–10,17,18], we hypothesized that LVAD recipients would show exercise gas exchange characterized by a high ventilation/perfusion ratio due to wasted ventilation of underperfused alveoli.

## Materials and methods

### Study population

Fifty-two consecutive LVAD recipients referred to our Institute for assessment of clinical status and rehabilitation were evaluated. Indication for LVAD implantation was hemodynamic decompensation due to ischemic or idiopathic HFrEF not responsive to intensive medical therapy. Inclusion criteria were: 1) clinical stability for 30 days prior to evaluation; 2) ability to carry out a cardiopulmonary exercise test; 3) possibility to take an arterial blood sample at rest and peak exercise; 4) respiratory exchange ratio ≤0.90 during arterial blood sampling at rest. Accordingly, 25 patients were enrolled. Exclusion of the remaining 27 patients was due to: lack of clinical stability and/or inability to carry out a cardiopulmonary exercise test (n. = 15, 29%), arterial blood sampling at rest and/or peak exercise not feasible (n. = 7, 13%) and resting respiratory exchange ratio >0.90 (n. = 5, 10%). Two groups of HFrEF patients with peak VO_2_ matched to that of LVAD or ≥14 ml/kg/min (HFrEF-matched and HFrEF≥14, respectively) were used as controls. Inclusion criteria for HFrEF were the same as those used for LVAD, with the addition of history of HFrEF from ischemic or idiopathic dilated cardiomyopathy and clinical/pharmacological stability for at least three months at the time of evaluation.

The protocol was approved by the Central Ethics Committee of the Salvatore Maugeri Foundation, IRCCS and informed written consent was obtained from all participants. The work has been carried out in accordance with The Code of Ethics of the World Medical Association (Declaration of Helsinki).

### Cardiopulmonary exercise test

Cardiopulmonary exercise test was carried out on an electromagnetic bicycle ergometer (Ergo-metrics 800S, Sensormedics, Yorba Linda, CA, USA), using a ramp incremental (5, 7 or 10 W/min) protocol at a pedaling rate of 60 revs/min. Respiratory gas exchange measurements were obtained breath-by-breath (Vmax29, Sensormedics) using a mouthpiece as patient/metabolic cart interface. Peak VO_2_ was the highest 30 s average VO_2_ value over the last minute of the exercise phase. The first ventilatory threshold (1^st^VT) was estimated by the V-slope and/or as the nadir of the VE vs. VO_2_ relationship and the second ventilatory threshold (2^nd^VT), when present, as the nadir of the VE vs. VCO_2_ relationship (16).

The slope of the ventilation vs. volume of exhaled carbon dioxide (VCO_2_) relationship (VE/VCO_2_ slope) was evaluated excluding, when present, its final nonlinear portion due to acidotic ventilatory drive. Blood pressure was measured at rest and every two minutes during the exercise and recovery phases; a manual cuff sphygmomanometer was used in HFrEF patients, whereas and automated electronic device (Dinamap, GE Healthcare, Waukesha, WI, USA) was used in LVAD recipients.

### Arterial blood gas analysis

Arterial blood was sampled at rest and during the last minute of exercise from a radial artery, and immediately analyzed for blood gases and hemoglobin content (GEM® Premier 4000, Instrumentation Laboratory, Lexington, MA, USA). Operators took note of the time elapsed from the start of the rest and exercise phases and arterial blood sampling, which allowed temporal alignment of arterial blood and ergospirometric data.

### Venous blood analysis

Fasting blood samples were drawn from an antecubital vein within 3±2 days prior to cardiopulmonary exercise testing. Blood samples (4 ml) were collected in EDTA vacutainers, and centrifuged at 3000 rpm at room temperature for 10 min. Plasma fractions (2–3 ml) were collected in appropriately labeled, sterile cryotubes, stored at -80°C and subsequently analyzed for blood urea nitrogen (BUN) by kinetic UV assay (Cobas® e 601, Roche Diagnostics US, Indianapolis, IN, USA), creatinine by Jeffé method (Cobas® e 601, Roche Diagnostics US) and N-terminal pro-brain natriuretic peptide (NT pro-BNP) by enzyme linked fluorescent assay (miniVIDAS®, bioMérieux SA, Marcy l’Etoile, FR). The BUN/creatinine ratio was calculated and used as a surrogate of circulating effective volume (Rose, 2000).

### Gas exchange

Ventilation/perfusion mismatch characterized by a high ventilation/perfusion ratio, i.e. by hypoperfusion of normally ventilated alveoli, determines an increase of physiological dead space (VD) and wasted ventilation. Arterial-end-tidal PCO_2_ difference (P(a-ET)CO_2_, mmHg) was used as a descriptor of alveolar wasted ventilation, and physiological dead space-tidal volume ratio (VD/VT) as an index of total wasted ventilation [14,16,19]. VD/VT was calculated as (16)

$$VD/VT=[(PaCO_{2}-PECO_{2})/PaCO_{2}]-VD_{m}/(VT-VD_{m})$$

where VT (l) is tidal volume, PaCO_2_ is arterial PCO_2_ (mm Hg, assumed to be equal to ideal alveolar PCO_2_), PĒCO_2_ (mm Hg) is mixed expired PCO_2_, equal to [VCO_2_ (l/min, STPD) / ventilation (l/min, STPD)] × (PB—47), and VDm (l) is breathing valve dead space (0.075 l).

Ventilation/perfusion mismatch characterized by a low ventilation/perfusion ratio, i.e. by hypoventilation of normally perfused alveoli and/or of diffusion limitation to gas exchange, is described by an increased alveolar-arterial PO_2_ difference (P(A-a)O_2_, mm Hg) [14,16,19]. Alveolar PO_2_ (PAO_2_, mm Hg) was calculated as (16)

$$PAO_{2}=[FIO_{2}\times (PB-47)]-PaCO_{2}/RER$$

where FIO_2_ is the fraction of inspired O_2_, dry, PB (mm Hg) is barometric pressure and RER is respiratory exchange ratio.

### Spirometry and lung diffusing capacity

Forced vital capacity and forced expiratory volume in 1 s were measured through a mass flow sensor (Vmax22, Sensormedics) according to the American Thoracic Society/European Respiratory Society criteria [20], and expressed as percentage of predicted values [21]. Lung diffusing capacity for carbon monoxide (DL_CO_) was measured using the single breath-hold maneuver following the recommendations of the American Thoracic Society [22] corrected for hemoglobin values [23] and reported as percentage of predicted values [24]. DL_CO_ was also expressed normalized for alveolar volume (DL_CO_/VA) [25].

### Echocardiography

Echocardiographic imaging (Vivid 7, General Electric Medical Systems, Milwaukee, WI, USA) was performed according to indications for LVAD recipients [26]. Special care was taken to measure tricuspid annular plane systolic excursion (TAPSE) and estimate pulmonary artery systolic pressure (PASP). The TAPSE/PASP ratio (mm/mmHg) was used as a surrogate of the *in vivo* right ventriculo-arterial coupling [27,28].

### Statistics

One-way analysis of variance with Fisher’s protected least significant difference post hoc tests, repeated-measure analysis of variance with post hoc contrasts and Fisher’s exact test were used to compare quantitative and qualitative variables, as appropriate. The association between continuous variables was explored by simple regression and Pearson product moment coefficients. Multiple regression was used to identify the independent predictors of peak P(a-ET)CO_2_ among a set of possible clinical/instrumental determinants of alveolar wasted ventilation. The level of statistical significance was a two-tailed p value of ≤0.05. The StatView® 5.0.1. (SAS Institute, Inc.; Cary, NC, USA) software package was used for statistical calculations.

## Results

### Demographic and clinical-instrumental characteristics

Study population demographic and clinical/instrumental characteristics are shown in Table 1. All patients were male. Study groups were well matched as to age and body mass index, with a predominance of ischemic origin of HFrEF and a markedly reduced left ventricular ejection fraction (LVEF). TAPSE and PASP were measurable in all patients. A stepwise significant increase of TAPSE from LVAD to HFrEF**≥**14 was observed, whereas PASP did not differ between the three groups; accordingly, TAPSE/PASP ratio progressively and significantly increased from LVAD to HFrEF**≥**14. Forced vital capacity, DL_CO_ and DL_CO_/VA did not differ between LVAD, HFrEF-matched and HFrEF**≥**14.

**Table 1:** Study population demographic and clinical/instrumental characteristics.

	LVAD	HfrEF-matched	HFrEF≥14	ANOVA and χ2 p
**n.**	25	15	15	/
**Age (yrs)**	61±5	63±9	62±6	0.60
**BMI (kg/m^2^)**	25.65±3.8	28.50±4.46	26.54±2.50	0.13
**Etiology, ischemic [n. (%)]**	17 (69)	11 (73)	15 (100)	0.69
**LVEF (%)**	27±8	30±6	32±5	0.059
**TAPSE (mm)**	13±2 **	18±4 *	21±5	<0.0001
**PASP (mmHg)**	37±12	38±8	34±8	0.67
**TAPSE/PASP (mm/mmHg)**	0.35±0.12 **	0.47±0.15 *	0.61±0.17	0.0049
**FVC %predicted**	74±16 *	77±14*	102±15	<0.0001
**DL_CO_ %predicted**	65±16	75±19	72±14	0.12
**DL_CO_/VA %predicted**	71±19	72±18	65±16	0.49
**Hb (g/dl)**	12.0±1.4 **	13.9±1.6	15.1±2.3	<0.0001
**BUN (mg/dl)**	44.0±24.0	61.3±28.7	46.9±20.3	0.12
**Creatinine (mg/dl)**	1.25±0.49	1.30±0.44	1.32±0.95	0.95
**BUN/Creatinine**	34.5±10.6 **	47.1±12.8	40.7±15.5	0.026
**NT pro-BNP (pg/ml)**	2341±1869	1880±1571	1137±1004	0.11
**β-blockers, yes [n. (%)]**	20 (80)	15 (100)	14 (87)	0.54
**Furosemide dosage (mg/day)**	60±64	104±87	43±40	0.09

LVAD = left ventricular assist device patients; HFrEF-matched = chronic heart failure patients with peak VO_2_ matched to that of LVAD; HFrEF≥14 = chronic heart failure patients with peak VO_2_ ≥14 ml/kg/min; ANOVA = analysis of variance; BMI = body mass index; LVEF = left ventricular ejection fraction; TAPSE = tricuspid annular plane systolic excursion; PASP = pulmonary artery systolic pressure; FVC %pred. = forced vital capacity as percentage of predicted; DL_CO_ %pred. = hemoglobin-adjusted lung diffusion capacity for carbon monoxide as percentage of predicted; DL_CO_/VA = hemoglobin-adjusted lung diffusion capacity for carbon monoxide normalized for alveolar volume as percentage of predicted; Hb = hemoglobin; BUN = blood urea nitrogen; NT pro-BNP = N-terminal pro-brain natriuretic peptide.

* = p <0.01 vs. HFrEF≥14

** = p <0.01 vs. HFrEF-matched and HFrEF≥14.

Analogously, BUN and creatinine were similar in the three study groups; of note, however, BUN/creatinine ratio was significantly lower in LVAD than in HFrEF-matched and HFrEF≥14. NT pro-BNP showed a progressive but non-significant decrease from LVAD to HFrEF≥14. BUN/creatinine ratio was not related with NT pro-BNP either in LVAD or in the whole study population (r = 0.04, p = 0.87 and r = 0.23, p = 0.31, respectively). All patients were similarly treated with beta-blockers, whereas a trend towards a higher dosage of loop diuretics in HFrEF-matched than in LVAD and HFrEF≥14 was detected.

### LVAD characteristics

Table 2 summarizes implanted device characteristics.

**Table 2:** Left ventricular assist device characteristics.

n.	25
**LVAD type [n. (%)]**	HM 15 (60)
	HW 10 (40)
**Indication [n. (%)]**	BT 3 (12)
	BD 5 (20)
	DT 17 (68)
**Time sinceimplantation (days)**	203±115
**Pump speed (rpm)**	HM 9242±450
	HW 2511±97
**Resting PBF (l/min)**	HM 4.6±0.8
	HW 4.1±0.7
**Peak PBF (l/min)**	HM 6.0±0.9
	HW 5.8±1.0

HM = HeartMate II; HW = HeartWare; BT = bridge to transplantation; BD = bridge to decision; DT = destination therapy; PBF = pump blood flow.

Slightly more than half of the patients were implanted with a HeartMate II, and destination therapy or bridge to decision was the indication to implantation in the majority of cases. Time from implantation averaged around seven months. Of note, even though pump speed was higher in HeartMate II than in HeartWare, both resting and peak pump blood flow, as measured by the device controller, were found to be similar in the two groups.

### Cardiopulmonary exercise testing

Cardiopulmonary exercise testing data are shown in Table 3. Resting parameters did not differ between the three study groups, with the only exception of resting systolic blood pressure that was significantly lower in LVAD than in the other two groups. By design, peak VO_2_ was lower in LVAD and HFrEF-matched than in HFrEF≥14, and the same was true for peak VCO_2_, peak ventilation, peak tidal volume, peak PETCO_2_, peak heart rate, peak work rate and VE/VCO_2_ slope. Conversely, peak respiratory rate did not differ between the three study groups, so differences in peak ventilation were mostly due to differences in peak tidal volume. No differences in 1^st^VT and 2^nd^VT detection were observed in the three study groups, so excluding possible influences of exercise-induced hyperventilation on exercise gas exchange. Peak respiratory exchange ratio was similar in the three groups and on average higher than 1.10, attesting maximal effort attainment in the whole study population. Peak O_2_ pulse was significantly lower in LVAD than in the other two groups, consistent with the acknowledged scarce contribution of the fixed-speed LVAD/dysfunctioning native left ventricle combination to systemic perfusion increase during exercise. Peak systolic blood pressure was not measurable in most LVAD due to technical problems, and did not differ between HFrEF-matched and HFrEF≥14. No differences were observed between the three study groups in the prevalence of exertional oscillatory ventilation, which, when detected, always disappeared in the second half of the exercise phase.

**Table 3:** Cardiopulmonary exercise testing parameters.

	LVAD	HFrEF-matched	HFrEF≥14	ANOVA
**Resting VO_2_ (l/min)**	0.225±0.059	0.232±0.60	0.254±0.068	0.33
**Peak VO_2_ (l/min)**	0.827±0.254	0.864±0.312	1.317±0.290 *	<0.0001
**Peak VO_2_ (ml/kg/min)**	10.6±1.7	10.8±1.3	17.8±2.8 *	<0.0001
**Peak VO_2_% predicted**	39±5	41±6	66±9 *	<0.0001
**Resting VCO_2_ (l/min)**	0.192±0.053	0.194±0.048	0.214±0.064	0.38
**Peak VCO_2_ (l/min)**	0.943±0.34347	0.985±0.366	1.501±0.308 *	<0.0001
**Resting VE (l/min)**	10.3±2.6	10.5±2.0	10.8±1.8	0.99
**Peak VE (l/min)**	42.1±7.7	44.2±4.4	53.4±11.4 *	0.024
**Resting Vt (l)**	0.567±0.120	0.548±0.131	0.608±0.136	0.41
**Peak Vt (l)**	1.423±0.281	1.420±0.287	1.847±0.383 *	0.0004
**Resting RR (breaths/min)**	18±5	19±3	18±5	0.63
**Peak RR (breaths/min)**	30±4	32±8	29±5	0.29
**1^st^VT, yes [n. (%)]**	19 (75)	12 (80)	15 (100)	0.19
**2^nd^VT, yes [n. (%)]**	15 (60)	11 (73)	12 (80)	0.27
**Resting RER**	0.83±0.04	0.81±0.04	0.83±0.05	0.40
**Peak RER**	1.14±0.08	1.13±0.07	1.14±0.06	0.90
**Resting PETCO (mmHg)**	31.1±4.3	31.4±3.6	33.3±3.3	0.11
**Peak PETCO_2_ (mmHg)**	27.8±3.7	29.4±3.9	33.1±4.9 *	0.0003
**Resting HR (beats/min)**	75±12	68±12	68±12	0.18
**Peak HR (beats/min)**	107±17	106±16	122±18 *	0.016
**Resting O_2_ pulse (ml/beat)**	3.0±0.9	3.4±1.1	3.6±1.4	0.24
**Peak O_2_ pulse (ml/beat)**	7.8±1.6 **	9.3±2.0	10.7±2.4	0.0002
**Resting SBP (mmHg)**	89±8 **	110±9	122±12	0.33
**Peak SBP (mmHg)**	/	141±13	157±10	0.39
**Peak work rate (W)**	62±14	75±22	108±24 *	<0.0001
**VE/VCO_2_ slope**	40.7±5.2	37.8±4.4	30.0±3.3 *	<0.0001
**EOV [n. (%)]**	6 (25)	4 (27)	2 (13)	0.11

LVAD = left ventricular assist device patients; HFrEF-matched = chronic heart failure patients with peak VO_2_ matched to that of LVAD; HFrEF≥14 = chronic heart failure patients with peak VO_2_ ≥14 ml/kg/min; ANOVA = analysis of variance; VE = ventilation; Vt = tidal volume; RR = respiratory rate; RER = respiratory exchange ratio; HR = heart rate; SBP = systolic blood pressure; 1^st^VT = first ventilatory threshold; 2^nd^VT = second ventilatory threshold; EOV = exercise oscillatory ventilation.

* = p <0.01 vs. LVAD and HFrEF-matched

** = p <0.01 vs. HFrEF-matched and HFrEF≥14.

Peak VO_2_ and VE/VCO_2_ slope were directly related to TAPSE/PASP (r = 0.56 and 0.49, respectively, both p <0.001) and LVEF (r = 0.32 and 0.45, respectively, both p <0.05). In LVAD, neither peak VO_2_ nor VE/VCO_2_ slope were related to time elapsed since implantation (r = 0.25 and 0.31, respectively, both p >0.25).

### Arterial blood gas analysis and ventilation/perfusion matching

Arterial blood gas analysis data are shown in Table 4. Resting and peak PO_2_ and resting and peak hemoglobin O_2_ saturation did not differ between the three study groups. Significantly higher resting and peak PCO_2_ values and lower peak pH values were detected in HFrEF≥14 than in LVAD and HFrEF-matched. Overall, the resting picture was consistent with normal arterial blood gas analysis with a slight trend towards respiratory alkalosis in LVAD and HFrEF-matched, whereas at peak exercise compensated (LVAD and HFrEF-matched) and partially compensated metabolic acidosis was evident (HFrEF≥14).

**Table 4:** Arterial blood gas analysis parameters.

	LVAD	HFrEF-matched	HFrEF≥14	ANOVA
**Resting PO**_**2**_ **(mmHg)**	85.8±9.4	85.5±10.9	85.8±9.1	0.97
**Peak PO**_**2**_ **(mmHg)**	91.5±15.4	93.5±13.3	98.3±9.2	0.37
**Resting SaO**_**2**_	0.98±0.01	0.98±0.01	0.97±0.01	0.55
**Peak SaO**_**2**_	0.98±0.02	0.98±0.02	0.97±0.01	0.91
**Resting PCO**_**2**_ **(mmHg)**	35.6±4.9	35.5±4.3	39.8±3.8 *	0.015
**Peak PCO**_**2**_ **(mmHg)**	32.1±4.1	33.2±3.6	35.9±4.2 *	0.017
**Resting pH**	7.44±0.03	7.43±0.03	7.40±0.02	0.39
**Peak pH**	7.41±0.05	7.40±0.04	7.35±0.05 *	0.001
**Resting HCO**_**3**_**- (mmol/l)**	24.1±2.9	24.2±2.2	24.8±1.8	0.57
**Peak HCO**_**3**_**- (mmol/l)**	21.1±2.8	21.4±2.2	20.8±2.3	0.35

LVAD = left ventricular assist device patients; HFrEF-matched = chronic heart failure patients with peak VO_2_ matched to that of LVAD; HFrEF≥14 = chronic heart failure patients with peak VO_2_ ≥14 ml/kg/min; ANOVA = analysis of variance; SaO_2_ = hemoglobin O_2_ saturation.

* = p <0.01 vs. LVAD and HFrEF-matched.

**Fig 1:**
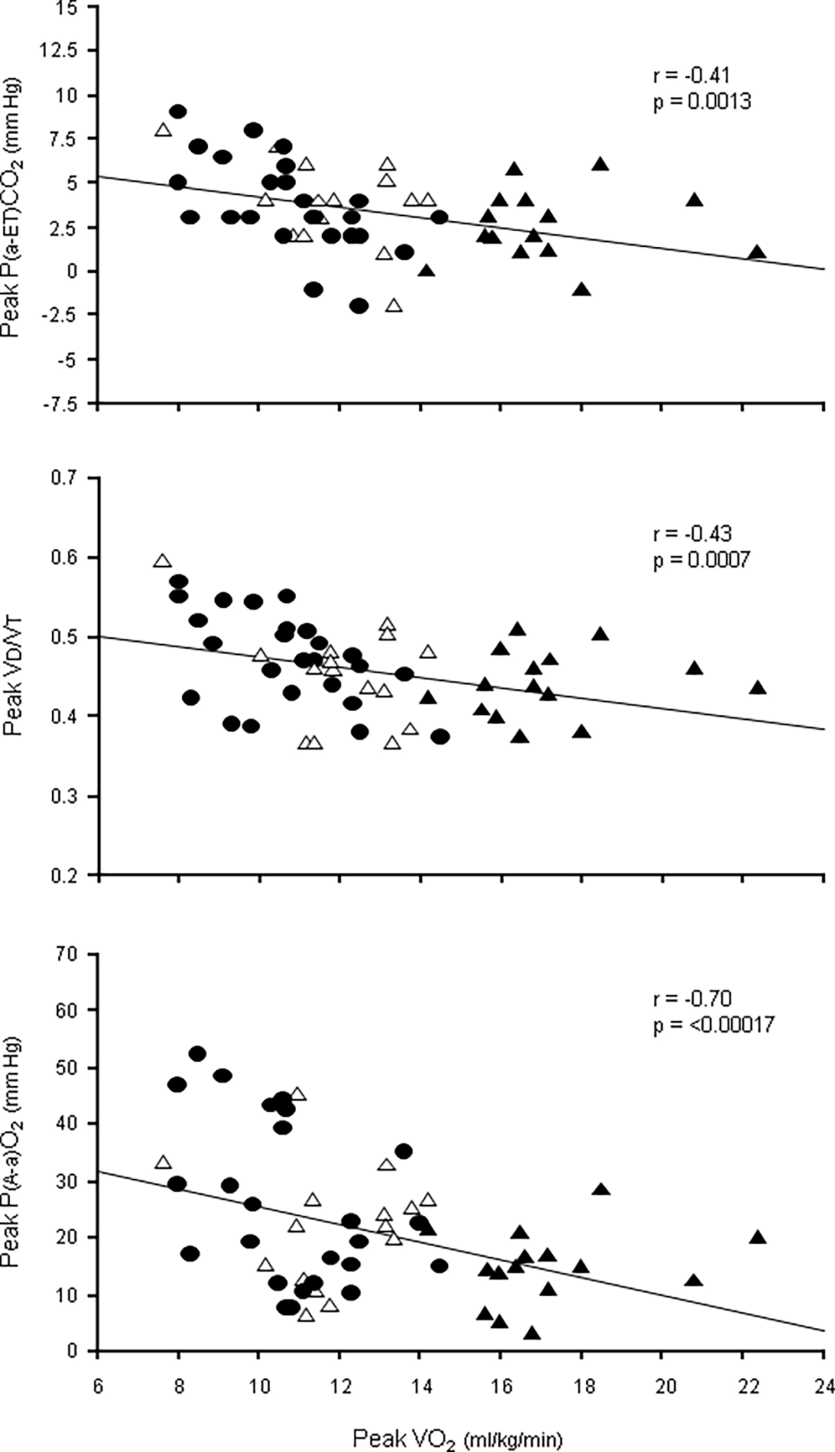
Peak arterial-end-tidal PCO_2_ difference, physiological dead space-tidal volume ratio and alveolar-arterial PO_2_ difference as a function of peak VO_2_ in the study population. The prevalence of high peak P(a-ET)CO_2_ and peak VD/VT values in the 3 study groups testify to a trend toward high ventilation/perfusion ratio mismatch in the whole study population. Full circles, empty triangles and full triangles are LVAD, HFrEF-matched and HFrEF≥14, respectively. P(a-ET)CO_2_ = arterial-end-tidal PCO_2_ difference; VD/VT = physiological dead space-tidal volume ratio; P(A-a)O_2_ = alveolar-arterial PO_2_ difference.

P(a-ET)CO_2_ at rest was normal in LVAD and HFrEF-matched and higher than normal in HFrEF≥14, and at peak exercise positive values were found in all groups (Table 5). Of note, a significant time [rest/peak exercise] × main effect [study group] interaction was detected (p = 0.001), indicating a significantly different rest-to-peak P(a-ET)CO_2_ change across the three study groups. Indeed, post hoc contrasts revealed no significant rest-to-peak change of P(a-ET)CO_2_ in LVAD and HFrEF-matched, whereas a significant decrease was observed in HFrEF≥14. Accordingly, peak P(a-ET)CO_2_ became more positive the more severe the exercise limitation was (Table 5 and Fig 1).

**Table 5:** Ventilation/perfusion matching parameters.

	LVAD	HFrEF-matched	HFrEF≥14	ANOVA
**Resting P(a-ET)CO**_**2**_ **(mmHg)**	4.5±2.4	4.1±1.4	6.5±3.6 *	0.046
**Peak P(a-ET)CO**_**2**_ **(mmHg)**	4.3±2.2	3.8±2.5	2.8±2.0 #	0.16
**Resting VD/VT**	0.51±0.04	0.51±0.04	0.49±0.07	0.85
**Peak VD/VT**	0.48±0.06	0.47±0.07	0.44±0.05	0.16
**Resting P(A-a)O**_**2**_ **(mmHg)**	16.4±9.3	13.9±7.4	12.4±7.8	0.35
**Peak P(A-a)O**_**2**_ **(mmHg)**	25.9±15.0 **	18.6±13.2	14.7±6.4	0.025

LVAD = left ventricular assist device patients; HFrEF-matched = chronic heart failure patients with peak VO_2_ matched to that of LVAD; HFrEF≥14 = chronic heart failure patients with peak VO_2_ ≥14 ml/kg/min; ANOVA = analysis of variance; P(a-ET)CO_2_ = arterial-end-tidal PCO_2_ difference; VD/VT = physiological dead space-tidal volume ratio; P(A-a)O_2_ = alveolar-arterial PO_2_ difference.

* = p <0.01 vs. LVAD and HFrEF-matched

** = p <0.01 vs. HFrEF≥14

# = p <0.01 vs. Resting P(a-ET)CO_2_

In LVAD, a multiple regression model including possible clinical/instrumental determinants of alveolar wasted ventilation (r = 0.99, r2 = 0.91, p <0.0001), selected TAPSE/PASP ratio and blood urea nitrogen/creatinine ratio as independent predictors of peak P(a-ET)CO_2_ (Table 6).

**Table 6:** Multiple regression testing independent predictors of peak arterial-end-tidal PCO_2_ difference in left ventricular assist device recipients.

	Coefficient	
TAPSE/PASP (mm/mmHg)	-0.60	0.042
BUN/Creatinine	-0.70	0.047
FVC %predicted	-0.075	0.29
Peak HR (beats/min)	-0.023	0.59
Time since implantation (days)	0.001	0.73
DL_CO_/VA %predicted	-0.081	0.18
Peak PBF (l/min)	-0.11	0.22
Peak RR (breaths/min)	-0.012	0.51
LVEF (%)	-0.058	0.47

TAPSE/PASP = tricuspid annular plane systolic excursion-pulmonary artery systolic pressure ratio; BUN = blood urea nitrogen; FVC %predicted = forced vital capacity as percentage of predicted; HR = heart rate; DL_CO_/VA %predicted = hemoglobin-adjusted lung diffusion capacity for carbon monoxide normalized for alveolar volume, expressed as percentage of predicted; PBF = pump blood flow; RR = respiratory rate; LVEF = left ventricular ejection fraction.

Higher than normal and similar resting VD/VT values were found in the 3 groups. A trend towards a larger rest-to-peak decrease of VD/VT was observed in HFrEF≥14 than in LVAD and HFrEF-matched, and peak VD/VT was found to be inversely related to peak VO_2_ (Fig 1) (Table 5).

Rest and peak P(A-a)O_2_ were normal in all groups (Table 5). However, peak P(A-a)O_2_ values were related to those of peak VO_2_ in the study population (Fig 1), and found to be higher in LVAD than in HFrEF≥14 (Table 5).

Finally, in LVAD no correlation was observed between P(a-ET)CO_2_, VD/VT and P(A-a)O_2_ and time elapsed since implantation (r between 0.18 and 0.35, all p >0.30).

## Discussion

Our results show that, notwithstanding the device support to cardiac function, LVAD-implanted patients still suffer from a severe exercise ventilation/perfusion mismatching, similar to that of patients with stable advanced HFrEF and independent of time elapsed from implantation. Such a pathophysiological picture is characterized by increased alveolar wasted ventilation due to hypoperfusion of normally ventilated alveoli and related to right ventriculo-arterial coupling and effective circulating volume.

### Pathophysiological background

LVAD implantation determines a peculiar exercise hemodynamic environment, where systemic perfusion can be generated in parallel by the LVAD and the native left ventricle [8,9,18]. Pump-generated blood flow can actually increase during exercise [17,18,29,30], but in any case quite modestly [8,17,18], as expected on the grounds of the reduced preload sensitivity of fixed-pump speed devices [10]. Some contribution of the native left ventricle can also become detectable, but is usually limited as well, ranging between 15 and 25% of peak systemic perfusion [8]. As a consequence, LVAD-implanted patients often suffer from a reduced functional capacity [4–8] due to the limited ability of the LVAD/dysfunctioning left ventricle combination to adapt to the increasing hemodynamic demands of exercise.

### LVAD recipients’ exercise gas exchange

**Fig 2:**
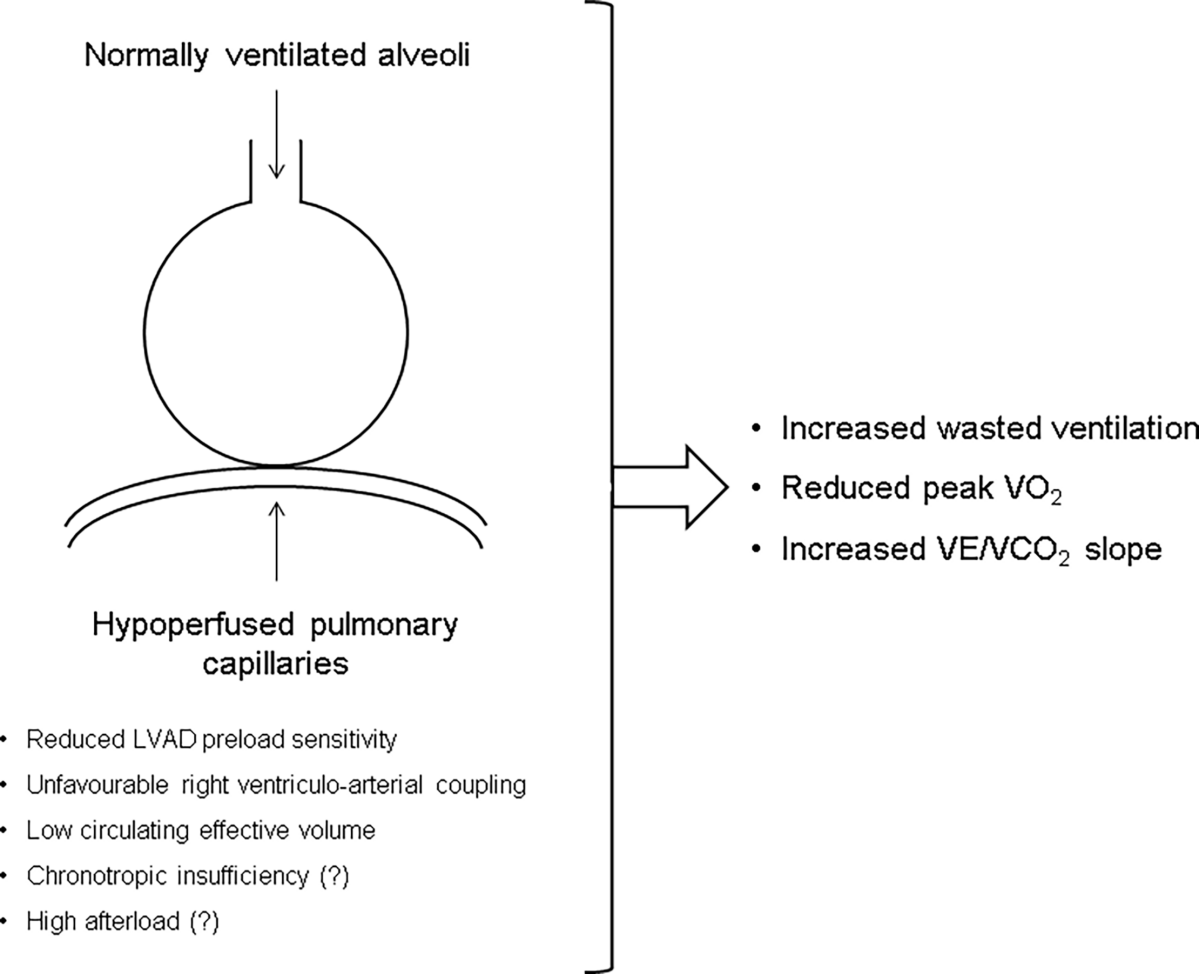
Schematic summarizing ventilation/perfusion pathophysiology in left ventricular assist device recipients. The scarce left ventricular assist device preload sensitivity constrains systemic perfusion increase during exercise causing hypoperfusion of normally ventilated alveoli, which in turn leads to an increase of wasted alveolar ventilation, ventilation/perfusion ratio and VE/VCO_2_ slope and to a reduction of peak VO_2_. This picture might be accentuated in patients with unfavorable right ventriculo-arterial coupling caused by impaired right ventricular contractility and/or increased pulmonary pressures, low circulating effective volume due to excessive diuretic use and possibly chronotropic insufficiency and increased afterload. LVAD = left ventricular assist device.

Resting P(a-ET)CO_2_ was normal in LVAD and HFrEF-matched and higher than normal in HFrEF≥14. The reason for the latter finding is not straightforward. P(a-ET)CO_2_ can increase with age, emphysema, hypovolemia and pulmonary embolism, but no evidence was available for differences as to these points in HFrEF≥14 as compared to the other two groups. High ventilation/perfusion ratio with increased wasted ventilation due to low cardiac output state is the obvious alternative cause of increased P(a-ET)CO_2_, but the higher peak VO_2_ observed by design in HFrEF≥14 than in LVAD and HFrEF-matched virtually rules out this point as well. Of note, however, in keeping with previously reported data in HFrEF [15], peak P(a-ET)CO_2_ was inversely related to peak VO_2_. Accordingly, a significant rest-to-peak decrease of P(a-ET)CO_2_ was found in HFrEF≥14, whereas LVAD and HFrEF-matched showed values almost unchanged compared to baseline. The rest-to-peak stability of positive peak P(a-ET)CO_2_ values observed in LVAD recipients and HFrEF-matched testifies to high alveolar wasted ventilation throughout exercise [31]. These results are conceptually supported by those of Matsumoto [32], who found a significant direct relationship between PETCO_2_ at respiratory compensation point and peak cardiac index in HFrEF patients with positive peak P(a-ET)CO_2_ values. Indeed, the rest-to-peak increase of pump-generated blood flow was modest in LVAD, averaging 1.5 l/min. As highlighted by Robertson [33], such a reduced systemic perfusion can result *per se* in a ventilation/perfusion ratio increase independently of any underlying degree of regional ventilation/perfusion mismatch. Our data showed an inverse relationship also between peak VD/VT and peak VO_2_, but rest-to-peak changes of VD/VT in the study groups did not reach statistical significance. This might be explained by the fact that changes in alveolar wasted ventilation are somewhat ‘diluted’ in those of total wasted ventilation [19]. Remarkably, multiple regression analysis identified TAPSE/PASP and blood urea nitrogen/creatinine ratio as independent predictors of peak P(a-ET)CO_2_ in LVAD recipients. TAPSE/PASP ratio has been used an *in vivo* descriptor of right ventriculo-arterial coupling [27,28]. The observation of a correlation between TAPSE/PASP ratio and both peak P(a-ET)CO_2_ and peak VO_2_ in LVAD extends previous data of our laboratory [8], and is consistent with the emerging evidence that right ventricular systolic function is a crucial determinant of functional capacity in several pathophysiological conditions [34–37]. This is even more conceivable in the light of the acknowledged crucial role played by right ventricular systolic function in the management of LVAD recipients both pre- and post-implantation [38]. The blood urea nitrogen/creatinine ratio is commonly used as a descriptor of circulating effective volume status [39]. In this regard, our finding of a blood urea nitrogen/creatinine ratio significantly lower in LVAD recipients than in HFrEF patients seems to describe a higher replenishment of circulation in the former group. It must be acknowledged that blood urea nitrogen/creatinine ratio can increase independently of circulating effective volume in HFrEF patients, due to neurohumoral activation-induced urea reabsorption at the collecting duct level [40]. However, blood urea nitrogen/creatinine ratio was not related with NT pro-BNP in the study population, notwithstanding a strong trend towards higher NT pro-BNP values (i.e. higher neurohumoral activation) in LVAD than in HFrEF patients. This justifies its use as a circulating effective volume descriptor in the present study. As a whole, these data favor the concept of right ventriculo-arterial coupling and repletion of systemic and pulmonary circulation as important factors in allowing for a proper ventilation/perfusion matching after LVAD implantation (Fig 2).

Similarly to previously reported data in HFrEF patients [15], peak P(A-a)O_2_ was found to be on average within normal limits in the study population, which should exclude the presence of low ventilation/perfusion ratio mismatching. However, LVAD had peak P(A-a)O_2_ values significantly higher than those of HFrEF-matched and HFrEF≥14. Agostoni [41] has shown that in advanced HFrEF patients alveolar-capillary membrane conductance is reduced shortly after incremental exercise, which could occur more likely in recently implanted patients. In addition, different responses of endothelial cells to laminar vs. oscillatory flow have been described [42,43], and how lack of pulsatile flow may affect alveolar-capillary membrane function in LVAD recipients has not been settled yet.

Of note, the time elapsed since implantation was not related to both ventilation/perfusion matching and peak VO_2_, which suggests quite a fixed exercise pathophysiological picture over time in LVAD recipients at least in the short- to medium-term after implantation. In this regard, the scarce adaptability of devices to exercise-induced preload increase is likely to play a preeminent role.

### Study limitations

Pump blood flow was estimated by device controllers based on pump speed and power consumption, and must be interpreted with some caution. We studied patients implanted with both axial- (HeartMate II) and centrifugal-flow (HeartWare) LVAD, known to show different sensitivities to changes in head pressure across the pump [9]. As LVAD peak blood pressure was not available in the present study, we cannot exclude that differences in afterload between patients may have influenced pump performance and exercise gas exchange. However, both resting and peak pump blood flow did not differ between patients implanted with HeartMate II and HeartWare devices, which should exclude at least large differences in afterload between the two groups. Ventilation/perfusion mismatch and wasted ventilation were considered the main determinants of the observed gas exchange abnormalities. However, it must be acknowledged that other factors not evaluated in the present study, such as abnormal pulmonary artery pressure increase or arterio-venous shunt during exercise can affect pulmonary gas exchange. In addition, the TAPSE/PASP ratio is a rough estimate of the *in vivo* right ventriculo-arterial coupling at rest; its role as a determinant of P(a-ET)CO_2_ at peak exercise must thus be interpreted with caution. Given that pulmonary gas exchange has been specifically evaluated in the present study, the concept of ‘hypoperfusion’ put forward in the manuscript applies only to pulmonary capillaries, thus not allowing inferences about gas exchange in the periphery, i.e. in exercising skeletal muscle [44]. Finally, we evaluated only male patients, so the behaviour of exercise gas exchange in female LVAD recipients remains to be determined.

## Conclusions

LVAD recipients still suffer from severely reduced functional capacity, similar to those of patients with stable advanced HFrEF. This study demonstrates that such a pathophysiological picture is mainly characterized by alveolar wasted ventilation and high ventilation/perfusion ratio due to hypoperfusion of normally ventilated alveoli, and is related to right ventriculo-arterial coupling and effective circulating volume. These results provide important insights into exercise pathophysiology of LVAD recipients, useful for the design of control algorithms able to adapt pump blood flow to exercise hemodynamic demands and yield a proper ventilation/perfusion matching.

## Supporting information

S1 Data file (XLS)
